# NFYA-Mediated TTK Up-Regulation Drives Fast Cell Cycle Progression and Its Inhibition Leads to Mitotic Catastrophe in Triple Negative Breast Cancer

**DOI:** 10.3390/cancers18091324

**Published:** 2026-04-22

**Authors:** Nianqiu Liu, Mengdi Zhu, Zijie Cai, Jingru Wang, Weihan Cao, Qianfeng Shi, Linghan Wang, Xiaoting Jiang, Jing Zhou, Jinna Lin, Wang Yang, Huipei Gan, Jianyun Nie, Qiang Liu

**Affiliations:** 1 Guangdong Provincial Key Laboratory of Malignant Tumor Epigenetics and Gene Regulation, Guangdong-Hong Kong Joint Laboratory for RNA Medicine, Breast Tumor Center, Sun Yat-sen Memorial Hospital, Sun Yat-sen University, Guangzhou 510120, China; 20201650@kmmu.edu.cn (N.L.); zhumd6@mail.sysu.cn (M.Z.); caizj@mail2.sysu.edu.cn (Z.C.); wangjr55@alumni.sysu.edu.cn (J.W.); shiqf3@mail.sysu.edu.cn (Q.S.); wanglh9@mail2.sysu.edu.cn (L.W.); jiangxt23@mail2.sysu.edu.cn (X.J.); jinxin@kmmu.edu.cn (J.Z.); linjn27@mail.sysu.edu.cn (J.L.); yangw93@mail.sysu.edu.cn (W.Y.); ganhp@mail2.sysu.edu.cn (H.G.); 2 Breast Surgery Ward 2, The Third Affiliated Hospital of Kunming Medical University, Yunnan Cancer Hospital, Kunming 650100, China; 3 Breast Tumor Center, Sun Yat-sen Memorial Hospital, Sun Yat-sen University, Guangzhou 510120, China; 4 Breast Cancer Center, Hubei Cancer Hospital, Tongji Medical College, Huazhong University of Science and Technology, Wuhan 430079, China; 5 The Department of Ultrasound, The First Affiliated Hospital of Kunming Medical University, Kunming 650032, China; 20240518@kmmu.edu.cn

**Keywords:** NFYA, TTK, TNBC, fast proliferation, spindle assembly checkpoint

## Abstract

Background/Objectives: Triple-negative breast cancer (TNBC) is frequently characterized by notably elevated Ki-67 expression, a hallmark of uncontrolled rapid cell-cycle progression. However, the underlying mechanisms remain unclear, leading to limited therapeutic options. Methods: In this study, hub gene was identified through integrated bioinformatic analysis of public datasets (TCGA-BRCA and METABRIC). Subsequent functional validation was performed both in vitro and in vivo using siRNA-mediated knockdown and small-molecule inhibitors. Phenotypic effects—including cell viability, cell cycle distribution, DNA synthesis, and clonogenic survival—were comprehensively assessed using MTT assays, flow cytometry, EdU, and colony formation assays. Protein-level changes were confirmed by Western blotting and immunohistochemistry (IHC). To dissect the transcriptional regulation of the key hub gene TTK, we first predicted potential upstream transcription factors using the JASPAR database; binding specificity was then validated through in silico motif analysis, luciferase reporter assays, and chromatin immunoprecipitation followed by quantitative PCR (ChIP-qPCR). Results: The mitotic kinase TTK is significantly overexpressed in TNBC compared with non-TNBC breast cancers. Notably, TTK overexpression exhibited a strong positive correlation with elevated Ki-67 indices and reduced overall survival in TNBC patients. Functional validation demonstrated that pharmacological or genetic inhibition of TTK effectively induced G2/M cell-cycle arrest and potently suppressed TNBC proliferation in both in vitro cell cultures and in vivo xenograft models. Mechanistically, TTK overexpression stems from enhanced transcriptional initiation driven by the transcription factor NFYA binding to the CCAAT box in the TTK promoter—an interaction newly identified here. Concurrently, TTK blockade disrupted spindle assembly checkpoint (SAC) signaling via BUB1B/MAD1L1 downregulation, triggering mitotic arrest and catastrophe. Conclusions: Collectively, these findings establish TTK as a key cell-cycle regulator driving TNBC proliferation. More importantly, targeting mitotic control through TTK inhibition represents an efficient strategy to impede the aberrantly fast cell cycle progression in TNBC.

## Simple Summary

Triple-negative breast cancer (TNBC) is often marked by very high Ki-67 expression—indicating rampant, uncontrolled cell division—but the drivers behind this fast proliferation remain poorly understood. Our study identifies the mitotic kinase TTK as a central factor: it is markedly overexpressed in TNBC (vs. non-TNBC), strongly correlates with elevated Ki-67 levels and worse patient survival, and functionally fuels tumor growth. We show that TTK overexpression is transcriptionally activated by the factor NFYA binding to the CCAAT box in the TTK promoter—a newly discovered regulatory mechanism. Importantly, inhibiting TTK induces G2/M arrest, disrupts the spindle assembly checkpoint (via BUB1B/MAD1L1 downregulation), triggers mitotic catastrophe, and potently suppresses TNBC growth in cells and animal models. These findings position TTK not only as a key biological driver and prognostic biomarker in TNBC, but also as a promising therapeutic target to halt its aberrantly accelerated cell cycle.

## 1. Introduction

Rapid cell proliferation is a hallmark of cancer [1]. Among all breast cancer subtypes, triple-negative breast cancer (TNBC) exhibits the highest proliferation rate. The median Ki-67 index in TNBC is 60% [2,3,4], significantly higher than the 10–20% in HR+/HER2− breast cancers. High Ki-67 in TNBC indicates that the majority of cancer cells are actively undergoing cell cycle progression. The mechanism underlying fast cell cycle progression in TNBC remains poorly understood. It is important to identify the drivers of fast cell cycle progression in TNBC, as they may serve as potential therapeutic targets.

The proliferative phenotype of TNBC is underpinned by profound regulation of cell-cycle control, particularly through the bypass of G1/S and G2/M checkpoints [5,6]. Cyclin-dependent kinase 4 and 6 (CDK4/6) inhibitors have revolutionized treatment paradigms for hormone receptor-positive breast cancer by targeting the RB–Cyclin D1 axis [7]; nevertheless, CDK4/6 inhibitors are only effective in HR+/HER2- breast cancer, but not in TNBC. In TNBC, frequent loss of RB [7], deletions in Cyclin D1 [6], and compensatory amplification of c-MYC, CDKN2A/p16INK4A or Cyclin E1/B [8,9,10] undermine G1/S checkpoint control and confer intrinsic resistance to CDK4/6 inhibition. Consequently, targeting the G1/S checkpoint is unlikely to yield effective control of TNBC proliferation. Evasion of this checkpoint shifts cellular dependence toward a hyperactivated G2/M transition, characterized by the overexpression of mitotic regulators such as PLK1 and Aurora kinases [11,12,13]. This shift also drives pathological up-regulation of Ki-67, which may be exacerbated by RB loss [14]. Together, these alterations create a feed-forward loop that sustains rapid proliferation. Ki-67, a proliferation marker peaking during the G2/M phase [15], not only reflects mitotic progression but also interacts with several mitosis-related genes and actively contributes to mitotic regulation [15,16]. This hyperactivation may represent a key driver of rapid proliferation in TNBC.

Rapid proliferation in TNBC is associated with mitotic dysregulation and aberrant Ki-67 overexpression, highlighting the critical need to clarify the molecular mechanisms driving this phenotype. This study shows that the dual-specificity protein kinase TTK is a pivotal regulator of cell cycle progression in TNBC, with expression levels strongly correlated with elevated Ki-67 and accelerated tumor growth. Mechanistic analyses revealed that TTK inhibition disrupted mitotic fidelity, resulting in defective cell division and the substantial suppression of tumor progression.

## 2. Methods and Materials

### 2.1. Bioinformatics Analysis

Datasets from the Cancer Genome Atlas (TCGA) and the Molecular Taxonomy of Breast Cancer International Consortium (METABRIC) were obtained from the UCSC Xena platform and cBioPortal, respectively (https://gdc.xenahubs.net and https://www.cbioportal.org/datasets, accessed 10 December 2021–6 March 2022). Differentially expressed genes (DEGs) with a fold-change ≥2 and a *p*-value < 0.05 were identified using the Limma package. Volcano plots and Venn diagrams were generated using OmicStudio and Hiplot tools. Gene Ontology (GO) enrichment analysis of candidate genes was performed using DAVID (https://davidbioinformatics.nih.gov/, accessed 10 December 2021–6 March 2022). Histograms and circle plots were created using R software v4.0.4. Pearson correlation coefficients were calculated to assess the association between genes and Ki-67 levels, with a coefficient >0.6 indicating a strong correlation. Protein–protein interaction (PPI) networks and hub gene identification were conducted using STRING v11.5 and Cytoscape v3.10.3. Gene Set Enrichment Analysis (GSEA) was utilized to identify signaling pathways enriched by differential gene expression analysis (TNBC vs. non-TNBC). Expression and survival prognosis of target genes were analyzed using the bc-GenExMiner v4.8 platform (http://bcgenex.ico.unicancer.fr, accessed 10 December 2021–6 March 2022).

### 2.2. Cell Culture and Transfection

The cell lines MCF-7 (accession number: HTB-22), T-47D (accession number: HTB-133), MDA-MB-231 (accession number: HTB-26), BT-549 (accession number: HTB-122), MDA-MB-468 (accession number: HTB-132), and 293T (accession number: CRL-3216) were obtained from the American Type Culture Collection (ATCC), SUM-149 (CL-0740) and SUM-159 (CL-0622) were purchased from Procell system (Wuhan, China). and cultured in Dulbecco’s Modified Eagle Medium (DMEM, Gibco Life Sciences (Gibco), C11995500BT, New York, NY, USA) supplemented with 10% fetal bovine serum (NEWZERUM, FBS-S500, Christchurch, New Zealand). For siRNA and plasmid transfections, cells were seeded at a density of 2 × 10^5^ cells/well in a 6-well plate. Transfections were conducted using specific siRNAs (100 nM, GenePharma, Shanghai, China) or plasmids (Kidan, Guangzhou, China) with Lipofectamine 3000 transfection reagent (ThermoFisher, L3000150, Waltham, MA, USA) following the manufacturer’s protocols. The sequences of all siRNAs used are provided below (Table 1).

### 2.3. Real-Time Quantitative Polymerase Chain Reaction (RT-qPCR)

Total RNA was extracted from breast cancer cells using the RNA-Quick Purification Kit (ESscience, RN001, Shanghai, China). The extracted RNA was reverse-transcribed into complementary DNA (cDNA) using the cDNA Synthesis Supermix (YEASEN, 11141ES60, Shanghai, China). RT-qPCR was performed using SYBR Green Master Mix (YEASEN, 11198ES08, Shanghai, China) in accordance with the manufacturer’s instructions. Amplification reactions were carried out using the LightCycler 480 system with gene-specific primers obtained from PrimerBank (https://pga.mgh.harvard.edu/primerbank/, accessed 10 December 2021–6 March 2022) and PrimerBlast (https://blast.ncbi.nlm.nih.gov/Blast.cgi, accessed 10 December 2021–6 March 2022). The sequences of all primers utilized in this study are listed below (Table 2).

### 2.4. Western Blot Analysis

Proteins were extracted from cells using RIPA Lysis Buffer (Beyotime, P0013B, Shanghai, China) supplemented with protease and phosphatase inhibitors (ThermoFisher, 78442, Waltham, MA, USA). Protein samples were separated by sodium dodecyl sulfate-polyacrylamide gel electrophoresis (SDS-PAGE) and subsequently transferred onto polyvinylidene difluoride (PVDF) membranes. Membranes were incubated overnight at 4 °C with primary antibodies, including, TTK (Signalway Antibody, 32677-2, 1:1000, Greenbelt, MD, USA), MAD1L1 (Proteintech, 18322-1-AP, 1:1000, Wuhan, China), BUB1B (Abcam, ab254326, 1:1000, Shanghai, China), CyclinB2 (Proteintech, 28603-1-AP, 1:1000), Caspase-2 (Cell Signaling Technology (CST), 2224T, 1:1000, Danvers, MA, USA), γ-H2AX (CST, 9718S, 1:1000, Danvers, MA, USA), NFYA (Proteintech, 12981-1-AP, 1:1000, Wuhan, China), and HRP-conjugated GAPDH (Proteintech, HRP-60004, 1:5000, Wuhan, China). Subsequently, membranes were incubated with horseradish peroxidase (HRP)-conjugated secondary antibodies (anti-mouse or anti-rabbit; CST, 7076/7074, 1:5000 dilution, Danvers, MA, USA). Protein-antibody complexes were visualized using an enhanced chemiluminescence (ECL) assay (Invitrogen, 34095, Waltham, MA, USA).

### 2.5. TTK Inhibitor Application

BAY-1217389 and AZ-3146 were procured from Selleck Chemicals (product codes S8215 and S2731, respectively, Houston, TX, USA). The half-maximal inhibitory concentration (IC50) and optimal treatment duration for TTK inhibitors across TNBC cell lines were determined using the MTT assay. Based on the calculated IC50 values, appropriate drug concentrations were selected for subsequent assays, including MTT, colony formation, EdU incorporation, and Western blot analyses. AZ-3146 was applied at concentrations of 0 µM, 1 µM, and 2 µM, while BAY-1217389 was used at concentrations of 0 nM, 2 nM, and 5 nM. The treatment duration was 96 h.

### 2.6. MTT, Colony Formation, and EdU Assays

MTT powder (Beyotime, ST316, Shanghai, China) was dissolved in sterile phosphate-buffered saline (PBS) to a final concentration of 5 mg/mL and applied to cells cultured in 96-well plates at a 1:10 volume ratio. After 4 h of incubation at 37 °C, absorbance was measured at 490 nm using a microplate spectrophotometer. For the colony formation assay, cells were seeded in 6-well plates at a density of 2000 cells/per well and incubated in medium. Subsequently, the cells were fixed with 4% paraformaldehyde and stained with crystal violet to facilitate colony counting. EdU proliferation assays were performed using an EdU Cell Proliferation Kit with Alexa Fluor 555 (Beyotime, C0075S, Shanghai, China), following the manufacturer’s instructions. Briefly, cells were seeded in 96-well plates at a density of 4000 cells/well and treated with siTTK for 48 h or TTK inhibitors for 96 h. Fluorescence microscopy (Olympus IX71, OLYMPUS, Tokyo, Japan) was used to capture images.

### 2.7. Flow Cytometry for Cell Cycle Analysis

Flow cytometry was utilized to evaluate cell cycle distribution using a CytoFLEX S flow cytometer (Beckman Coulter, Suzhou, China). Cells, exceeding one million in number, were fixed in 75% cold ethanol for over 24 h and subsequently stained with 100 μL of propidium iodide (PI) solution, including 50 μL of RNAase. The stained cells were analyzed by flow cytometry. And gating strategy was applied using ModFit v3.1.0.0 to exclude cell debris and aggregates, ensuring accuracy.

### 2.8. Animal Experiments

All animal experiments were conducted with the approval of the South China University of Technology Laboratory Animal Research Center (AEC NO. 2022042). Four-week-old BALB/C nude mice were obtained from the animal research center, and housed in a specific pathogen-free environment under a 12 h light/dark cycle in temperature- and humidity-controlled cages, with ad libitum access to food and water. MDA-MB-231 cells were implanted into the mammary fat pads of the mice, and once tumors become palpable, the experimental groups were administered oral doses of BAY-1217389 (5 mg/kg) or AZ-3146 (10 mg/kg), respectively. Mouse body weight was recorded at the start of the experiment, and tumor growth was monitored daily. Tumor sizes were measured every three days. When tumor volumes approached 1500 mm^3^, all mice were humanely euthanized, and final tumor volumes were measured and recorded. Excised tumors were subsequently processed for immunohistochemical staining.

### 2.9. Patient and Tumor Specimens

Paraffin-embedded tumor samples were obtained from 203 postoperative tumor tissue specimens collected between 2016 and 2022 from breast cancer patients at the Breast Tumor Center of Sun Yat-sen Memorial Hospital who had not received neoadjuvant or other therapy. The cohort included 11 cases of DCIS, 30 cases of Luminal A breast cancer, 29 cases of Luminal B breast cancer, 29 cases of HER2-overexpressing breast cancer, and 104 cases of TNBC. Patients were classified as a missing or deceased event prior to 1 January 2022 (1) and as a visited or alive event after 1 January 2022 (0). All samples were collected with signed informed consent and in accordance with the guidelines of the internal review and ethics boards of Sun Yat-sen Memorial Hospital.

### 2.10. Immunohistochemical Analysis

Paraffin-embedded tissue sections prepared by Servicebio were processed as follows: sections were heated at 65 °C for 2 h to facilitate antigen retrieval, followed by dewaxing in xylene for 10 min and dehydration through a graded series of alcohol (100%, 95%, 85%, 75%, and 50%) for a total of 25 min. Subsequently, the sections were boiled in 4× EDTA antigen retrieval solution (pH 9.0, ZSBIO, ZLI-9069, Beijing, China) for 15 min and allowed to cool to room temperature. They were then incubated for 10 min with 3% endogenous peroxidase blocking solution (BOSTER, AR1108, Wuhan, China). Following this, the sections were incubated in PBS containing 5% bovine serum albumin (BSA) for 30 min, and then incubated overnight at 4 °C with primary antibodies against TTK (HuaBio, HA500249, 1:250, Hangzhou, China), NFYA (Proteintech, 12981-1-AP, 1:250, Wuhan, China), BUB1B (HuaBio, ER1802-76, 1:250, Hangzhou, China), MAD1L1 (Proteintech, 18322-1-AP, 1:250, Wuhan, China), and Ki-67 (ZSBIO, ZM0166, Beijing, China, ready to use). On the following day, the sections were incubated with corresponding secondary antibodies for 2 h at room temperature. Immunodetection was performed using DAB (ZSbio, ZLI-9017, Beijing, China) according to the manufacturer’s instructions, followed by hematoxylin staining of cell nuclei. Staining scores were assigned based on intensity: 0 (no staining), 1 (light brown), 2 (brown), and 3 (dark brown).

### 2.11. Immunofluorescence

Cells were seeded at a density of 1 × 10^5^ cells/well in confocal dishes and treated as per the experimental protocols. The cells were fixed with 4% paraformaldehyde for 15 min, followed by membrane permeabilization with PBS containing 0.5% Triton X-100 for 10 min and blocking with 1% BSA in PBST for 1 h. Primary antibodies against α-tubulin (Proteintech, 66031-1-IG, 1:250) and γ-tubulin (Proteintech, 15176-1-AP, 1:250) were applied for overnight incubation at 4 °C. On the following day, cells were incubated with corresponding fluorescent secondary antibodies, including anti-mouse (CST, 4408S, 1:1000, Danvers, MA, USA) and anti-rabbit (CST, 4413S, 1:1000, Danvers, MA, USA), for 2 h at room temperature. Nuclear staining was performed with DAPI for 10 min. Finally, the cells were imaged and captured using a confocal microscope (Leica TCS SP8 STED 3X, Leica Microsystems, Wetzlar, Germany).

### 2.12. Dual Luciferase Assay and Chromatin Immunoprecipitation

Cells were plated in 24-well plates at a concentration of 1 × 10^4^ cells/well and transiently transfected with 500 ng of the target plasmid and 10 ng of the TK plasmid per well. After a 48 h incubation, the Dual Luciferase Reporter Gene Assay Kit (Beyotime, RG027) protocol was followed. Subsequent assays were performed using a multifunctional microplate reader (TECAN, Spark10M, Männedorf, Switzerland). MDA-MB-231 cells were cultured in 15 cm oversized dishes until they reached full confluency, after which they were processed according to the SimpleChIP Plus Enzymatic Chromatin IP Kit (Cell Signaling Technology, CST, 56383S) instructions. The ChIP-grade antibody against NFYA (sc-17753X, 1:200, Santa Cruz, CA, USA) was employed, and the qPCR primers (Table 3) were designed accordingly.

Select the upstream 2000 bp promoter region of TTK transcription >NC_000006.12:80002649-80004648 Homo sapiens chromosome 6, GRCh38.p14 Primary Assembly. Cut out −2000~0, −1500~0, −1000~0, −500~0, −300~0, −50~0, and synthesize corresponding plasmids by Guangzhou IGE Biotechnology Co., Ltd. (Guangzhou, China).

### 2.13. Statistical and Survival Prognostic Analysis

Data visualization and *p*-value computation were performed using GraphPad Prism v9. For comparisons across multiple groups, Dunnett’s multiple comparison test following ordinary one-way analysis of variance (ANOVA), and Tukey’s multiple comparisons test following two-way ANOVA were applied. Comparisons between control and experimental groups were conducted using an unpaired *t*-test. All experiments were independently repeated at least three times with independent samples, and results were presented as means ± standard deviation (SD). Results with a *p*-value < 0.05 were considered statistically significant.

Survival curves were plotted using GraphPad Prism v9, including 104 TNBC cases and 99 non-TNBC cases. The *p*-values and hazard ratios were calculated using the log-rank (Mantel–Cox) and Mantel–Haenszel tests, respectively.

## 3. Results

### 3.1. Identifying TTK as a Key Regulator of Fast Proliferation in TNBC

**Figure 1:**
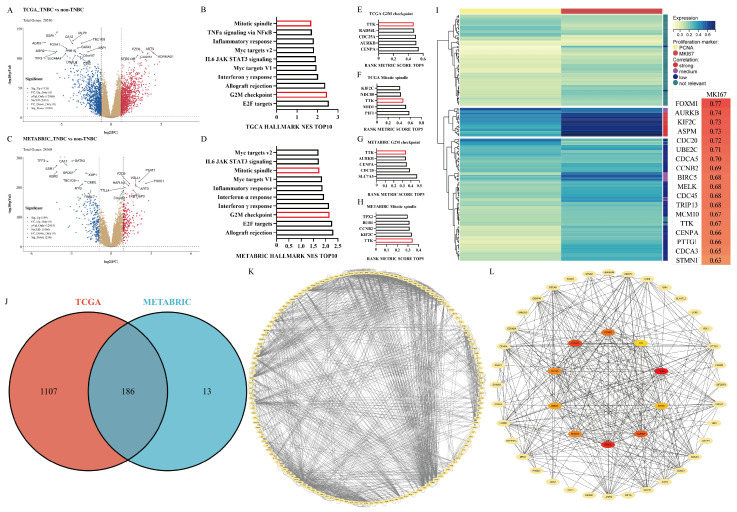
Bioinformatic analysis identifies TTK as a key regulator of proliferation in TNBC. (**A**,**E**) Volcano plots depicting differentially expressed genes (DEGs) between TNBC and non-TNBC samples ((**A**): TCGA database; (**E**): METABRIC database). (**B**–**D**,**F**–**H**) Gene set enrichment analysis (GSEA) results for DEGs, showing normalized enrichment score (NES) and rank metric score ((**B**–**D**): TCGA; (**F**–**H**): METABRIC). (**J**) Venn diagram illustrating the number of significantly upregulated genes common to both TCGA and METABRIC databases. (**K**) Protein–protein interaction (PPI) network of the 186 candidate genes. (**L**) Hub genes identified from the PPI network using the Maximal Clique Centrality (MCC) algorithm.

To investigate the molecular drivers underlying rapid proliferation in TNBC, transcriptomic data from the Cancer Genome Atlas (TCGA) and METABRIC cohorts were systematically analyzed. Comparative profiling between TNBC and non-TNBC tumors identified 1293 and 199 significantly up-regulated genes in TCGA and METABRIC, respectively (log2 fold-change ≥ 1, *p* < 0.05, Figure 1A,C). Gene set enrichment analysis (GSEA) of Hallmark pathways consistently revealed marked dysregulation of cell cycle-related processes, with the G2/M checkpoint and mitotic spindle pathways ranking among the top 10 enriched terms based on the normalized enrichment score (NES) in both datasets (Figure 1B,D). Leading-edge analysis further pinpointed TTK as the sole common gene implicated in both the G2/M checkpoint and mitotic spindle pathways (Figure 1E–H). Cross-cohort integration revealed 186 consistently up-regulated genes shared between TCGA and METABRIC (Figure 1J). Subsequent protein–protein interaction (PPI) network analysis, using the Matthews correlation coefficient (MCC) algorithm, identified TTK as a high-confidence hub, ranking among the top ten central nodes within the interaction network (Figure 1K,L).

Clinical relevance was further validated through correlation analyses with Ki-67 proliferation indices. Among the 186 candidate genes, TTK exhibited a strong positive correlation with this well-established marker of tumor aggressiveness (Figure 1I).

Taken together, these multi-dimensional analyses—encompassing differential gene expression, pathway enrichment, network centrality, and clinical correlation—collectively implicate TTK as a critical regulator of mitotic fidelity and proliferation capacity in TNBC. This integrated evidence supports the prioritization of TTK as a mechanistically and clinically relevant therapeutic target in this aggressive breast cancer subtype.

### 3.2. TTK Serves as a Prognostic Biomarker and Therapeutic Target in TNBC

**Figure 2:**
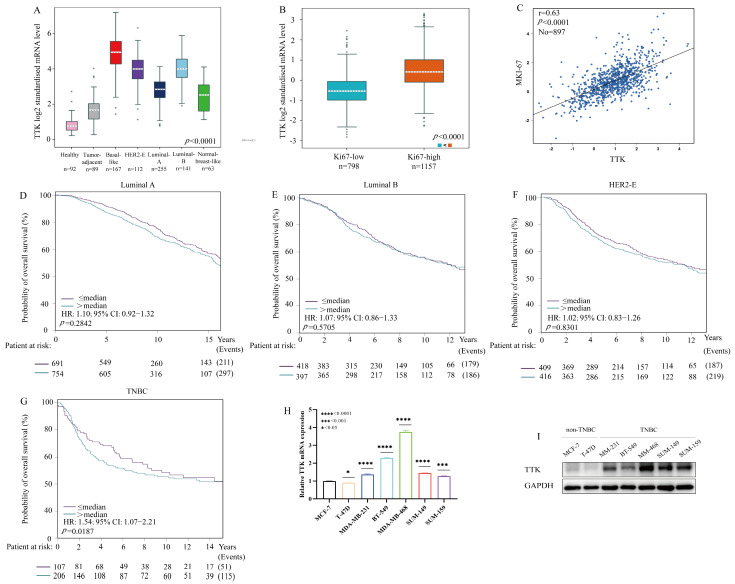
TTK is specifically upregulated in TNBC cell lines and associated with poor prognosis exclusively in TNBC patients. (**A**) Expression of TTK in normal tissues, adjacent tissues, and different breast cancer subtypes. (**B**) Expression of TTK across Ki−67 expression subgroups. (**C**) Correlation between TTK and MKI67 expression in TNBC. (**D**–**G**) Kaplan−Meier curves showing overall survival based on TTK expression in Luminal A (**D**), Luminal B (**E**), HER2−E (**F**) and triple negative (**G**) breast cancer. (**H**,**I**) mRNA (**H**) and protein (**I**) expression levels of TTK in various breast cancer cell lines. * < 0.05, *** < 0.001, **** < 0.0001. The uncropped blots are shown in Supplementary Materials.

**Figure 3:**
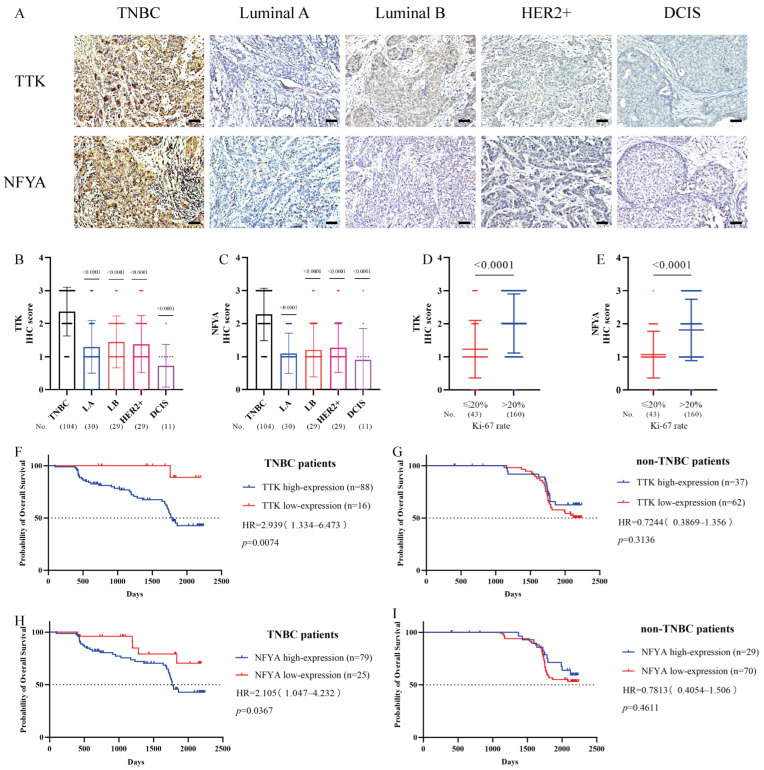
High specific expression of NFYA and TTK in TNBC tumor tissue, associated with poor prognosis in TNBC patients. (**A**) Immunohistochemistry (IHC) analysis of the expression of NFYA and TTK in various breast cancer tissues (scale bar = 50 μm). (**B**–**E**) IHC scores for NFYA and TTK in different breast cancer subtypes and Ki-67 expression subgroups. (**F**,**G**) Correlation between TTK expression and survival prognosis in TNBC and non-TNBC patients. (**H**,**I**) The correlation between NFYA expression and the survival prognosis in TNBC and non-TNBC patients.

TTK was identified as a critical regulator of proliferation in TNBC. Analysis using the bc-GenExMiner v4.8 database revealed that basal-like/TNBC tumors express the highest level of TTK mRNA among all breast cancer subtypes (Figure 2A). Consistently, this transcriptional profile was corroborated at both the cellular and tissue levels: TNBC cell lines displayed elevated TTK mRNA and protein levels compared with luminal/HER2+ lines (Figure 2H,I), and immunohistochemical staining further confirmed intensified TTK expression in TNBC tissues (Figure 3A,B).

The clinical relevance of TTK was reinforced by its strong association with cellular proliferation. A robust positive correlation was observed between TTK expression and Ki-67 indices (Figure 2C), with the highest TTK levels observed in tumors exceeding 20% Ki-67 positivity (Figure 2B and Figure 3D). Importantly, the prognostic significance of TTK was restricted to specific subtypes. High TTK expression predicted markedly worse overall survival in TNBC patients (Figure 2G and Figure 3F), whereas no significant association was observed in luminal or HER2+ subtypes (Figure 2D–F and Figure 3G).

In summary, we identifies TTK as a key driver of proliferation and a subtype-specific prognostic marker in TNBC. These findings provide compelling evidence supporting the therapeutic targeting of TTK, as a promising strategy for managing this aggressive malignancy.

### 3.3. TTK Drives the Rapid Proliferation of TNBC Cells by Safeguarding Mitotic Fidelity via Control of the Spindle Assembly Checkpoint (SAC)

**Figure 4:**
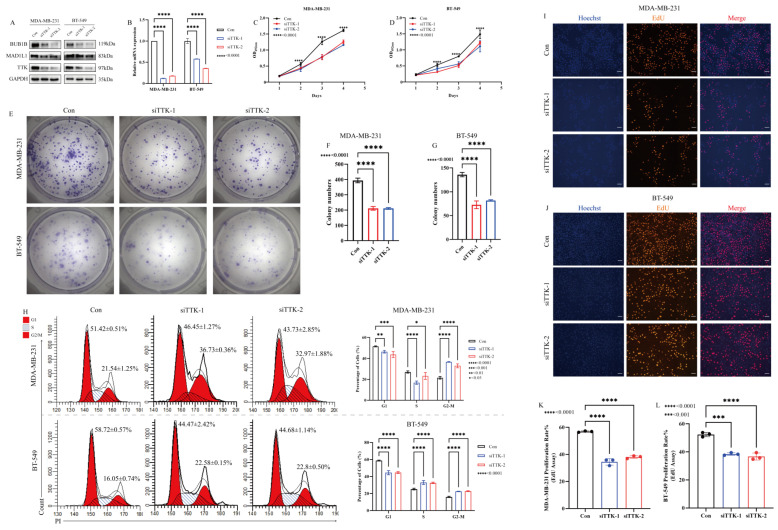
TTK knockdown inhibits TNBC cell proliferation by inducing M-phase arrest. (**A**) Changes in TTK, MAD1L1, and BUB1B protein expression. (**B**) Changes in TTK mRNA expression. (**C**,**D**) Proliferation curves. (**E**–**G**) Changes in colony formation ability. (**H**) Cell cycle distribution changes. (**I**–**L**) Changes in EdU assay. * < 0.05, ** < 0.01, *** < 0.001, **** < 0.0001. The uncropped blots are shown in Supplementary Materials.

To elucidate the role of TTK in cell-cycle regulation, both loss- and gain-of-function studies were conducted in TNBC cell lines. Small interfering RNA (siRNA)-mediated knockdown of TTK in TNBC cell lines (MDA-MB-231, BT-549) effectively reduced its mRNA and protein levels (Figure 4A,B). Functionally, TTK depletion significantly inhibited cell proliferation (Figure 4C,D), reduced clonogenic formation (Figure 4E–G), and induced G2/M arrest (Figure 4H). Mechanistically, the anti-proliferative effects were linked to SAC dysfunction, as TTK knockdown led to the downregulation of the critical SAC mediators MAD1L1 and BUB1B (Figure 4A). EdU incorporation assays further confirmed diminished DNA synthesis activity in TTK-deficient TNBC cells (Figure 4I–L).

Conversely, TTK overexpression accelerated proliferation and facilitated the G2/M transition in MCF-7 cells (Supplementary Figure S1A–F). Collectively, these reciprocal models demonstrate that TTK serves as a key molecular regulator of mitotic fidelity in TNBC, with its expression levels governing SAC integrity, cell-cycle dynamics, and the proliferation of tumor cells.

### 3.4. TTK Inhibition Disrupts SAC Function and Suppresses Proliferation in TNBC

To assess the therapeutic relevance of TTK inhibition in TNBC, two selective small molecule inhibitors, BAY-1217389 and AZ-3146, were evaluated across multiple TNBC models. Both inhibitors exerted dose-dependent anti-proliferative in various TNBC cell lines (MDA-MB-231, BT-549, SUM-149, and SUM-159; Supplementary Figures S2A–D, S3A–D, S4A–D and S5A–D), accompanied by marked suppression of clonogenic potential (Supplementary Figures S2E–G, S3E–G, S4E–G and S5E–G). Cell-cycle analysis revealed profound G2/M arrest following treatment (Supplementary Figures S2H, S3H, S4H and S5H), consistent with EdU assay results showing reduced DNA synthesis (Supplementary Figures S2I–L, S3I–L, S4I–L and S5I–L). Among the two compounds, BAY-1217389 exhibited superior potency. Mechanistically, both inhibitors reduced TTK expression in a dose-dependent manner and downregulated its core SAC effectors, BUB1B and MAD1L1 (Supplementary Figures S2M, S3M, S4M and S5M), confirming on-target disruption of mitotic control.

**Figure 5:**
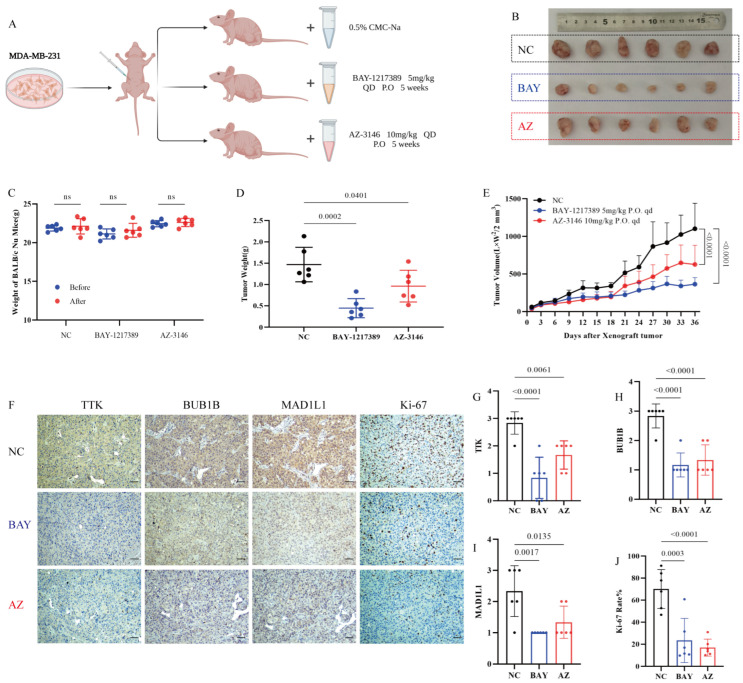
Effect of TTK inhibitors on MDA-MB-231 xenograft growth in BALB/c nude mice. Tumor representative images (**A**,**B**) and growth curves (**E**) showing tumor volume changes across treatment groups during the experiment. Body weight (**C**) and tumor weight (**D**) measurements of mice at experimental endpoint. Immunohistochemical (IHC) analysis of expression changes in tumor tissues (scale bar = 50 μm): representative staining images (**F**), TTK (**G**), BUB1B (**H**), MAD1L1 (**I**) and Ki-67 (**J**).

In vivo, administration of BAY-1217389 or AZ-3146 significantly reduced tumor growth in MDA-MB-231 xenograft models compared with controls (Figure 5A,B,E), with concordant decreases in tumor mass (Figure 5D). Immunohistochemical analysis (Figure 5F) confirmed TTK suppression (Figure 5G), loss of SAC effectors (BUB1B and MAD1L1, Figure 5H, 5I), and decreased Ki-67 expression in treated tumors (Figure 5J), with no evidence of systemic toxicity (Figure 5C).

Collectively, these results demonstrate that TTK inhibitors exert potent anti-tumor effects by disabling SAC-mediated mitotic activity and impairing proliferative capacity, underscoring the therapeutic potential of TTK as a critical target in cell cycle control for fast-proliferating TNBC.

### 3.5. TTK Depletion Induces Mitotic Catastrophe via SAC Collapse and DNA Damage Accumulation

**Figure 6:**
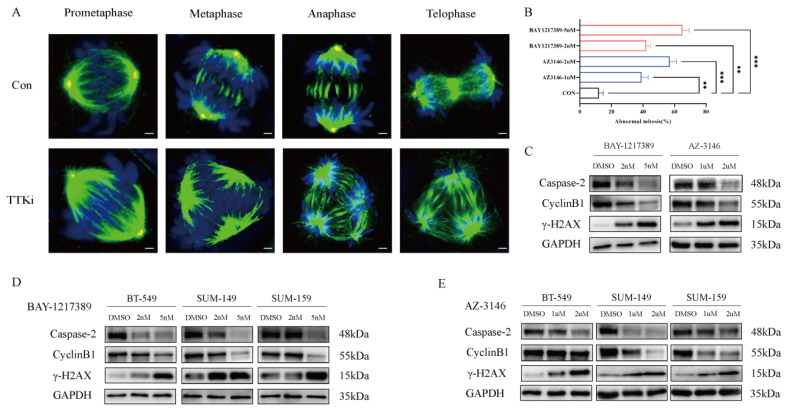
Targeted TTK inhibition impairs mitotic catastrophe and DNA damage. (**A**) Morphological changes during mitotic phases (prometaphase, metaphase, anaphase, telophase) and microtubule organization in MDA-MB-231 treated with a TTK inhibitor. Fluorescence staining: DAPI (blue, nuclei), α-tubulin (green, spindle microtubules), γ-tubulin (red, centromeres). Scale bar = 25 μm. (**B**) Percentage of MDA-MB-231 cells exhibiting abnormal mitosis. (**C**) Alterations in Caspase-2, Cyclin B1, and γ-H2AX protein expression in MDA-MB-231. (**D**,**E**) Alterations in Caspase-2, Cyclin B1, and γ-H2AX protein expression in other TNBC cell lines. ** < 0.01, *** < 0.001. The uncropped blots are shown in Supplementary Materials.

It is well-established that mitosis disruption can result in defective chromosomal segregation, leading to cell death characterized as mitotic catastrophe [17]. Consistent with this, TTK inhibitors induced hallmark features of mitotic failure in MDA-MB-231 cells, including multinucleation and aberrant division patterns (Figure 6A), with phenotypic severity increasing in a dose-dependent manner (Figure 6B). At the molecular level, SAC collapse was associated with genomic instability, as indicated by elevated γ-H2AX levels and the depletion of key SAC components (TTK, MAD1L1, and BUB1B, Supplementary Figures S2–S5M) and DNA repair mediators, including CCNB2 and Caspase-2 (Figure 6C–E).

This cascade—SAC disruption leading to chromosome mis-segregation, replication stress, and the accumulation of lethal DNA damage—underpins the anti-proliferative potency of TTK inhibition. These findings establish TTK as a critical regulator of mitotic fidelity in TNBC cells, whose inhibition results in SAC destabilization and drives cells into catastrophic mitotic failure.

### 3.6. Transcription Factor NFYA Engagement with the CCAAT-Box Site Promotes TTK Transcription and Translation in TNBC

**Figure 7:**
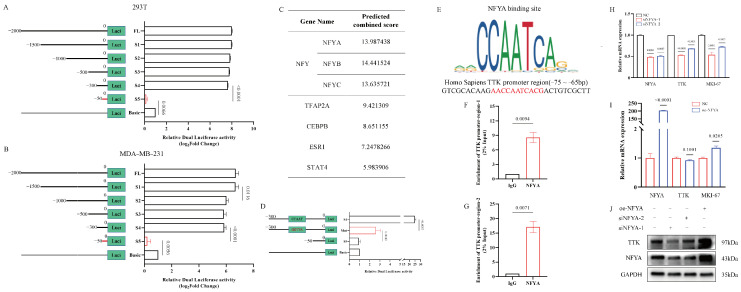
NFYA binding to CCAAT site regulates TTK expression (**A**,**B**) Effects of TTK promoter regions of varying lengths on transcription initiation. (**C**) Prediction of transcription factors and their binding scores using the JASPAR database. (**D**) Fluorescence intensity changes before and after mutation in the CCAAT binding site, as detected by dual-luciferase assay. (**E**) Base composition of the CCAAT site region within the TTK DNA promoter. (**F**,**G**) ChIP-qPCR analysis of NFYA binding to the TTK promoter DNA region (**H**–**J**) Alterations in NFYA and TTK mRNA and protein expression following NFYA knockdown or overexpression. The uncropped blots are shown in Supplementary Materials.

In our investigation of potential regulatory mechanisms for TTK, we focused on the promoter region of the TTK gene, which contains essential nucleotides for transcription initiation. Truncation analysis of this promoter region (−50~−300) (Figure 7A,B), revealed that the interaction between Nuclear Factor Y (NFY) and the CCAAT-box site plays a pivotal role in regulating TTK expression (Figure 7C). Bioinformatics analysis demonstrated a significant up-regulation of NFYA expression in TNBC, particularly in subgroups with high Ki-67 levels, as shown (Supplementary Figure S6A–C). Notably, lower NFYA expression was correlated with better survival outcomes in TNBC patients (Supplementary Figure S6D–G).

Subsequent experiments showed that mutations in the CCAAT-box significantly reduced fluorescence intensity, suggesting that NFYA directly binds to this specific site (Figure 7D–G). These findings suggest that NFYA acts as a direct regulator of TTK transcription and translation (Figure 7H–J). Supporting these in vitro results, analysis of clinical tumor samples revealed high levels of NFYA and TTK expression in TNBC and subgroups with high Ki-67 expression (Figure 3A–E). Crucially, increased expression of NFYA and TTK was associated with poorer prognosis in TNBC patients (Figure 3F–I). Collectively, these results present the first known link between NFYA and TTK, indicating that NFYA-mediated up-regulation of TTK drives fast proliferation in TNBC and may represent a potential therapeutic target.

## 4. Discussion

The transition of quiescent cells into a proliferative state is governed by the coordinated integration of extracellular ligand sensing, receptor-mediated signaling, and intracellular kinase cascades [18]. This signaling convergence proceeds through phosphorylation-dependent activation of CDK complexes—specifically CDK4/6-Cyclin D1—which sequentially phosphorylate downstream effectors to drive irreversible cell-cycle commitment [6]. Although CDK4/6 inhibitors targeting this axis have revolutionized the treatment of hormone receptor-positive breast cancer, their clinical efficacy in TNBC remains limited due to intrinsic molecular heterogeneity and frequent activation of compensatory pathways. The present study sought to address this therapeutic gap by identifying TTK, a mitotic checkpoint kinase, as a TNBC-enriched driver of proliferation. These findings establish TTK as both a TNBC-selective proliferative vulnerability and a predictive biomarker, defining a novel therapeutic avenue for this aggressive malignancy and providing a framework for targeting previously uncharacterized drivers of proliferation in precision oncology.

TTK (also known as Mps1) is a dual-specificity kinase encoded at chromosome 6q13-q21 [19], capable of phosphorylating both serine/threonine and tyrosine residues. During interphase (G1, S, G2 phases), TTK localizes to nuclear pore complexes and redistributes to kinetochores upon mitotic entry, where it mediates spindle microtubule attachment through centromere-associated protein E (CENPE) interaction—a process critical for ensuring accurate chromosome segregation [20,21]. In addition to its canonical role in mitotic regulation, TTK also maintains centrosome duplication and chromosomal stability [22,23]. Under physiological conditions, TTK expression is largely restricted to germline (testicular) and placental tissues [24]. However, in pathological states, it is aberrantly overexpressed across multiple malignancies, where it correlates with aneuploidy and genomic instability [25,26,27,28,29,30,31]. Based on integrative analyses, including transcriptomic, functional, and immunohistochemical data, our findings revealed a significant role for TTK overexpression in TNBC. Elevated TTK levels were associated with accelerated proliferation and adverse clinical outcomes, implicating this kinase as a core driver of the aggressive growth phenotype of TNBC. Although the present findings establish TTK as a TNBC-specific proliferative vulnerability, critical mechanistic questions remain—particularly those related to its mitosis-specific activity transitions. Addressing these mechanistic gaps is essential for the rational development of TTK-targeted therapies and may expand the precision oncology toolkit for overcoming cell-cycle-driven progression in TNBC.

We first investigated the mechanism underlying the markedly elevated expression of TTK in triple-negative breast cancer (TNBC). Gene expression is tightly regulated at both transcriptional and translational levels. Transcription initiation represents a critical early step and is subject to precise control by transcription factors that maintain cellular homeostasis during development [32,33]. These proteins bind to promoter regions upstream of coding sequences to regulate the timing and magnitude of gene expression [34].

In this study we conducted truncation and point mutation assays on the TTK promoter region, confirming that TTK transcription initiation is dependent on CCAAT motifs. Bioinformatic analysis using JASPAR revealed strong predicted binding affinities between members of the NF-Y family and these motifs—implicating them as key regulators of TTK transcription. Previous studies have demonstrated that NF-Y controls genes involved in G2/M phase transition; supporting rapid cellular proliferation [35,36,37]. Functionally active NF-Y complexes require all three subunits, with a reduction in NFYA being the most effective in disrupting CCAAT box binding [38,39,40,41]. Chromatin immunoprecipitation (ChIP) assays confirmed a direct interaction between NFYA and the CCAAT motif within the TTK promoter region. Regulation of NFYA resulted in concordant changes in both TTK mRNA (by RT-qPCR) and protein (by Western blot). Similar trends were observed for MKI-67 mRNA expression. Notably, TNBC tissues exhibited strikingly high levels of NFYA, and a robust correlation was observed between NFYA abundance (mirroring TTK expression) and clinical outcomes in TNBC patients.

Collectively, these findings demonstrate that NFYA directly targets the CCAAT site within the TTK promoter region driving specific up-regulation at both transcriptomic and proteomic levels. This explains the distinct overexpression of TTK observed in TNBC.

This investigation highlights the central role of TTK in sustaining TNBC progression through the subversion of the mitotic checkpoint. Mechanistically, TTK depletion induces mitotic arrest by destabilizing the mitotic checkpoint complex (MCC), a multiprotein assembly composed of BUB1B:BUB3 and MAD1L1:MAD2L1 heterodimers, which are essential for SAC signaling [42]. Disintegration of the MCC, characterized by the downregulation of BUB1B and MAD1L1, results in defective chromosome segregation and aberrant mitosis. To correct these errors and restore proper chromosome alignment, key regulators such as Aurora B and TTK/Mps1 [43,44] are required to reactivate SAC signaling. TTK plays a pivotal role in this process by recruiting BUB1B and MAD1L1 to the kinetochore, facilitating the subsequent assembly of BUB3 and MAD2L1. This hierarchical recruitment is essential for maintaining MCC stability and restoring SAC signaling [42,45,46,47]. Notably, TNBC cells exhibit elevated rates of centrosome amplification and chromosomal instability compared with other breast cancer subtypes [48], resulting in a heightened reliance on SAC surveillance mechanisms to ensure mitotic fidelity. This dependence underscores the critical role of SAC signaling in sustaining genomic instability in TNBC [49]. Our findings demonstrate that TTK inhibition disrupts this error correction machinery, transforming chromosomal instability from a survival mechanism into a therapeutic vulnerability.

Accurate chromosome segregation during mitosis is essential for maintaining genomic integrity and is tightly regulated by the SAC, a surveillance mechanism that delays anaphase onset until all kinetochores are properly attached to microtubules. Disruption of the SAC leads to mitotic catastrophe, an irreversible state characterized by proliferative arrest and apoptosis [17,50]. In TNBC, inherent chromosomal instability exacerbates this vulnerability—aneuploid tumor cells exhibit higher rates of mitotic errors compared with their diploid counterparts [51], with multinucleation (≥3 nuclei/per cell) serving as both a morphological marker and a functional driver of genomic instability [52]. Mechanistically, SAC failure triggers a cascade involving multinucleation and replication stress, as evidenced by mitotic catastrophe and the accumulation of γ-H2AX, a marker of DNA damage [51,53]. This response is further intensified by Caspase-2 depletion [54], which contributes to additional DNA damage and the downregulation of Cyclin B1 [55]. Loss of Cyclin B1 disrupts the mitotic checkpoint complex by eliminating the MAD1 binding platform required for SAC reactivation [56,57,58]. TTK inhibition compromises SAC integrity by destabilizing kinetochore-localized BUB1B-MAD1L1 complexes, which are essential for correcting kinetochore–microtubule attachment errors during the prometaphase–metaphase–anaphase transition. This checkpoint collapse results in irreversible mitotic arrest through two converging mechanisms: (1) mitotic catastrophe, characterized by chromosome missegregation, multinucleation, and chromatin bridging, and (2) DNA damage accumulation marked by γ-H2AX. By targeting TNBC-specific vulnerabilities in mitotic fidelity, TTK inhibition therapy transforms chromosomal instability from a survival advantage into a therapeutic liability.

## 5. Conclusions

In conclusion, our study identifies TTK as a critical regulator of fast cell-cycle progression in triple-negative breast cancer. TTK is significantly overexpressed in TNBC compared with other breast cancer subtypes and is strongly correlated with high Ki-67 levels and poor patient outcomes. For the first time, we demonstrate that NFYA-mediated transcriptional activation at the CCAAT motif within the TTK promoter drives this overexpression. Inhibition of TTK induces mitotic arrest and effectively suppresses TNBC proliferation both in vitro and in vivo. Mechanistically, TTK inhibition disrupts spindle assembly checkpoint signaling by downregulating BUB1B and MAD1L1, leading to mitotic catastrophe and DNA damage (Graphic Abstract). These findings establish TTK as a pivotal regulator of mitotic dynamics in TNBC and underscore its potential as a therapeutic target for controlling unchecked proliferation characteristic of this aggressive cancer subtype.

## Figures and Tables

**Table 1:** siRNA sequence.

siRNA	Sequence 5′-3′
siTTK-1	GGAUUUAAGUGGCAGAGAATT
siTTK-2	GGUCGUUACAGUCAAGCAATT
siNFYA-1	GAGCAGUAUACAGCAAACATT
siNFYA-2	GGAGGCCAGCUAAUCACAUTT

**Table 2:** qPCR primer sequence.

Primer	Forward (5′-3′)	Reverse (5′-3′)
TTK	GTGGAGCAGTACCACTAGAAATG	CCCAAGTGAACCGGAAAATGA
NFYA	CAGTGGAGGCCAGCTAATCAC	CCAGGTGGGACCAACTGTATT
MKI67	ACGCCTGGTTACTATCAAAAGG	CAGACCCATTTACTTGTGTTGGA
GAPDH	GGAGCGAGATCCCTCCAAAAT	GGCTGTTGTCATACTTCTCATGG

**Table 3:** qPCR primer sequence including CCAAT-region.

Primer	Forward (5′-3′)	Reverse (5′-3′)
TTK-P1	CTCCCAGGCAAAAATTCGGC	AAGCGACAGTCGTGATTGGT
TTK-P2	TCACTGGGTAGGTTTGCTCG	CGTAGAAGCGACAGTCGTGA

## Data Availability

The METABRIC dataset was downloaded from the cBioPortal for Cancer Genomics (https://www.cbioportal.org/study/summary?id=brca_metabric, accessed 10 December 2021–6 March 2022). The TCGA-BRCA RNA-seq (STAR counts) dataset was retrieved from the UCSC Xena Browser (https://xenabrowser.net/datapages/?dataset=TCGA-BRCA.star_counts.tsv&host=https%3A%2F%2Fgdc.xenahubs.net, accessed 10 December 2021–6 March 2022). NFYA and TTK expression profiles, along with associated clinical and survival data for breast cancer patients, were obtained from the Breast Cancer Gene-Expression Miner v4.8 (https://bcgenex.ico.unicancer.fr/BC-GEM/GEM-Accueil.php?js=1, accessed 10 December 2021–6 March 2022).

## Supplementary Materials

The following supporting information can be downloaded at: https://www.mdpi.com/article/10.3390/cancers18091324/s1, The uncropped blots; Supplementary Figure S1: TTK over-expression promote MCF-7 cell proliferation. (**A**) Changes in TTK mRNA expression; (**B**) Changes in TTK protein expression; (**C**) Proliferation curves; (**D**–**F**) Cell cycle distribution especially in mitosis progression. Supplementary Figure S2: AZ-3146 inhibits MDA-MB-231 and BT-549 cell proliferation. (**A**,**B**) IC50 values; (**C**,**D**) Proliferation curves; (**E**–**G**) Changes in colony formation ability; (**H**) Cell cycle distribution changes; (**I**–**L**) Changes in EdU assay (scale bar = 50 μm); (**M**) Changes in TTK, MAD1L1, and BUB1B protein expression. Supplementary Figure S3: BAY-1217389 inhibits MDA-MB-231 and BT-549 cell proliferation. (**A**,**B**) IC50 values; (**C**,**D**) Proliferation curves; (**E**–**G**) Changes in colony formation ability; (**H**) Cell cycle distribution changes; (**I**–**L**) Changes in EdU assay; (**M**) Changes in TTK, MAD1L1, and BUB1B protein expression. Supplementary Figure S4: AZ-3146 inhibits SUM-149 and SUM-159 cell proliferation. (**A**,**B**) IC50 values; (**C**,**D**) Proliferation curves; (**E**–**G**) Changes in colony formation ability; (**H**) Cell cycle distribution changes; (**I**–**L**) Changes in EdU assay; (**M**) Changes in TTK, MAD1L1, and BUB1B protein expression. Supplementary Figure S5: BAY-1217389 inhibits SUM-149 and SUM-159 cell proliferation. (**A**,**B**) IC50 values; (**C**,**D**) Proliferation curves; (**E**–**G**) Changes in colony formation ability; (**H**) Cell cycle distribution changes; (**I**–**L**) Changes in EdU assay; (**M**) Changes in TTK, MAD1L1, and BUB1B protein expression. Supplementary Figure S6: NFYA is specifically upregulated in TNBC and associated with poor prognosis exclusively in TNBC patients. (**A**) NFYA expression in healthy, tumor-adjacent, and tumor tissues; (**B**) NFYA expression in non-basal-like and basal-like breast cancer; (**C**) TTK expression across Ki-67 expression subgroups; (**D**–**G**) Kaplan-Meier survival curves illustrating overall survival based on TTK expression in various breast cancer subtypes.
