# Supplementary material for: NFYA-Mediated TTK Up-Regulation Drives Fast Cell Cycle Progression and Its Inhibition Leads to Mitotic Catastrophe in Triple Negative Breast Cancer
Source: Cancers (Basel). 2026 Apr 22;18(9):1324. doi: 10.3390/cancers18091324 (PMC13163004; doi:10.3390/cancers18091324)
Supplement: Supplementary file 1 [file cancers-18-01324-s001.zip › supplementary figures.docx]

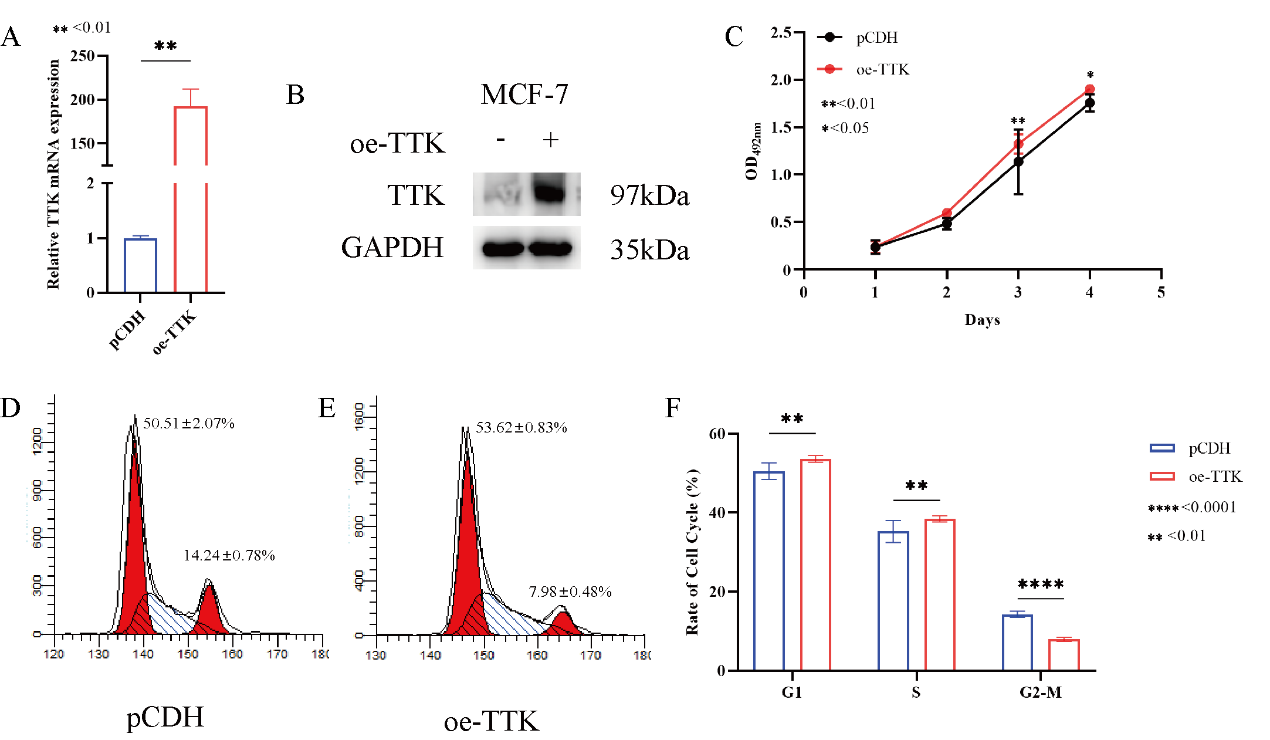


**Supplementary Figure S1.** TTK over-expression promote MCF-7 cell proliferation.

1. Changes in TTK mRNA expression; (B) Changes in TTK protein expression; (C) Proliferation curves; (D–F) Cell cycle distribution especially in mitosis progression.


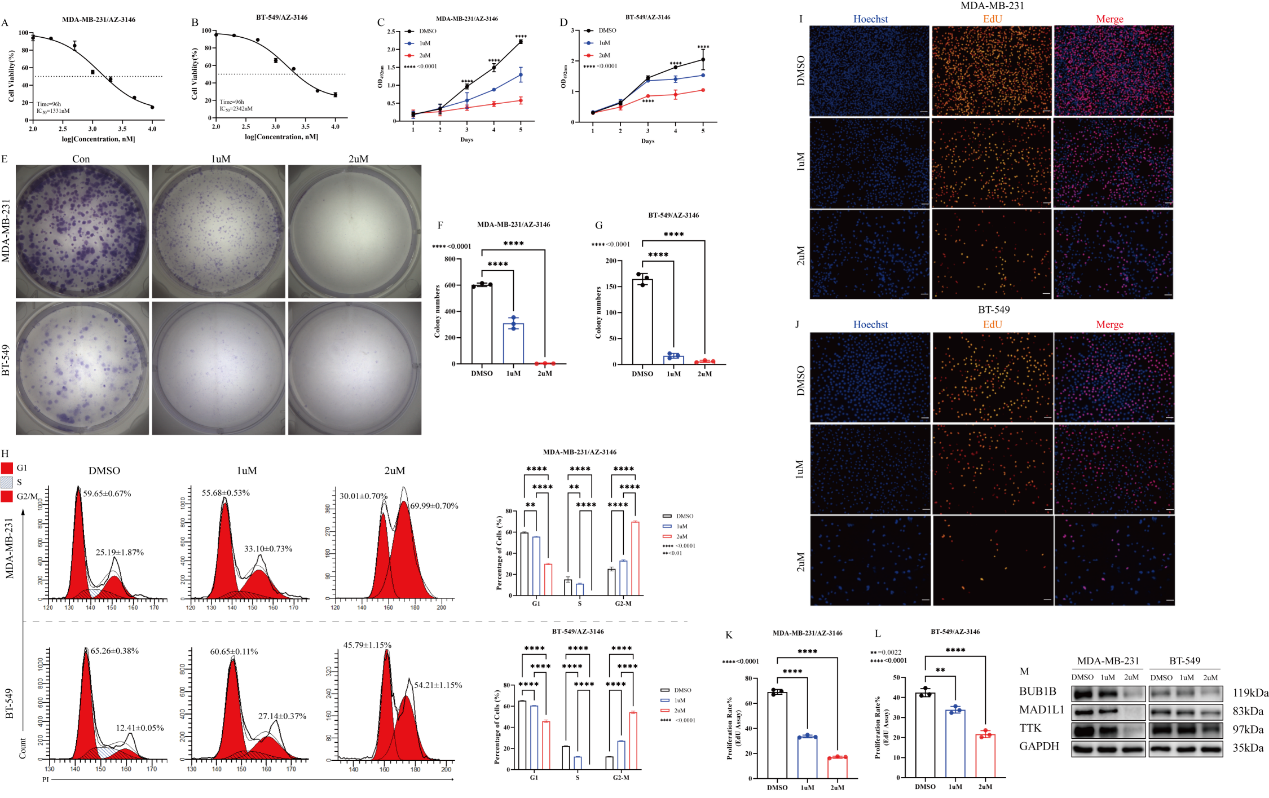


**Supplementary Figure S2.** AZ-3146 inhibits MDA-MB-231 and BT-549 cell proliferation.

(A, B) IC_50_ values; (C, D) Proliferation curves; (E–G) Changes in colony formation ability; (H) Cell cycle distribution changes; (I–L) Changes in EdU assay (scale bar=50μm); (M) Changes in TTK, MAD1L1, and BUB1B protein expression.


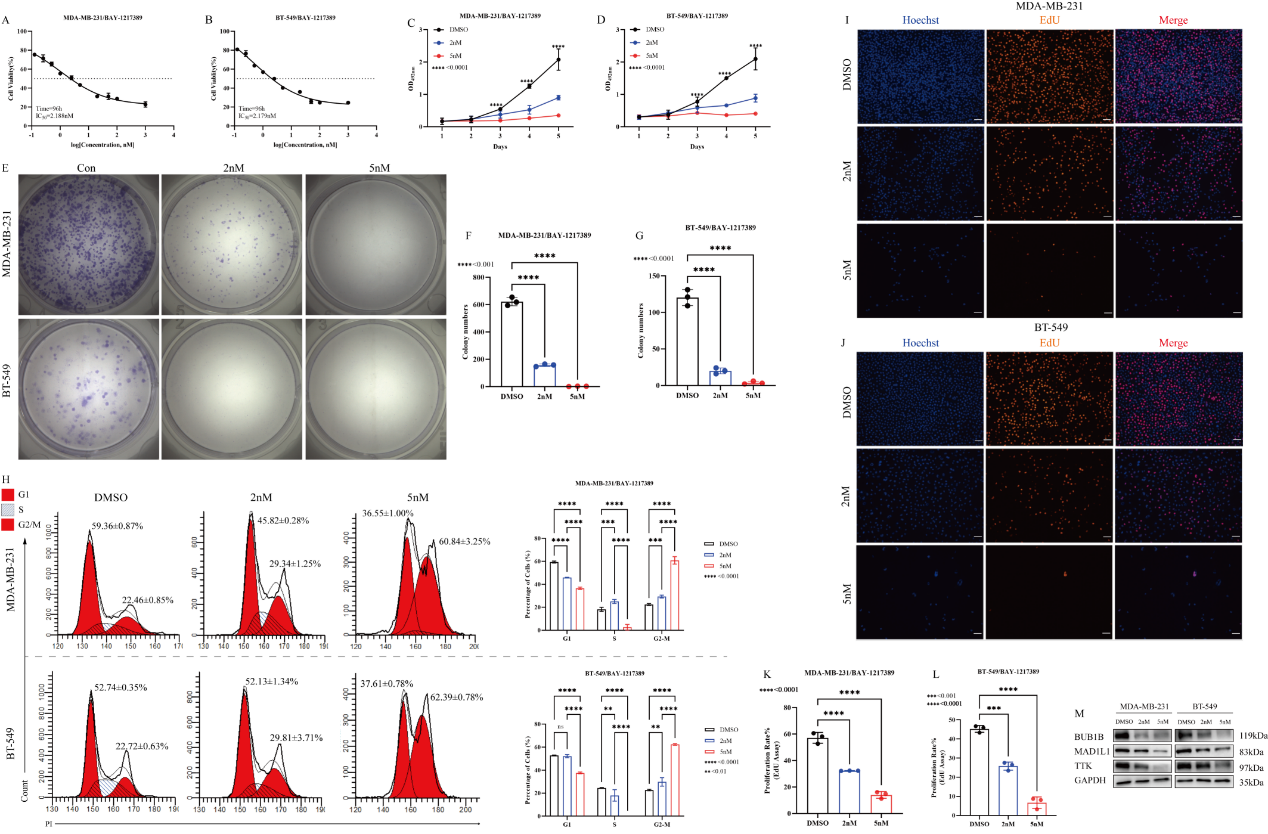


**Supplementary Figure S3.** BAY-1217389 inhibits MDA-MB-231 and BT-549 cell proliferation.

(A, B) IC_50_ values; (C, D) Proliferation curves; (E–G) Changes in colony formation ability; (H) Cell cycle distribution changes; (I–L) Changes in EdU assay; (M) Changes in TTK, MAD1L1, and BUB1B protein expression.


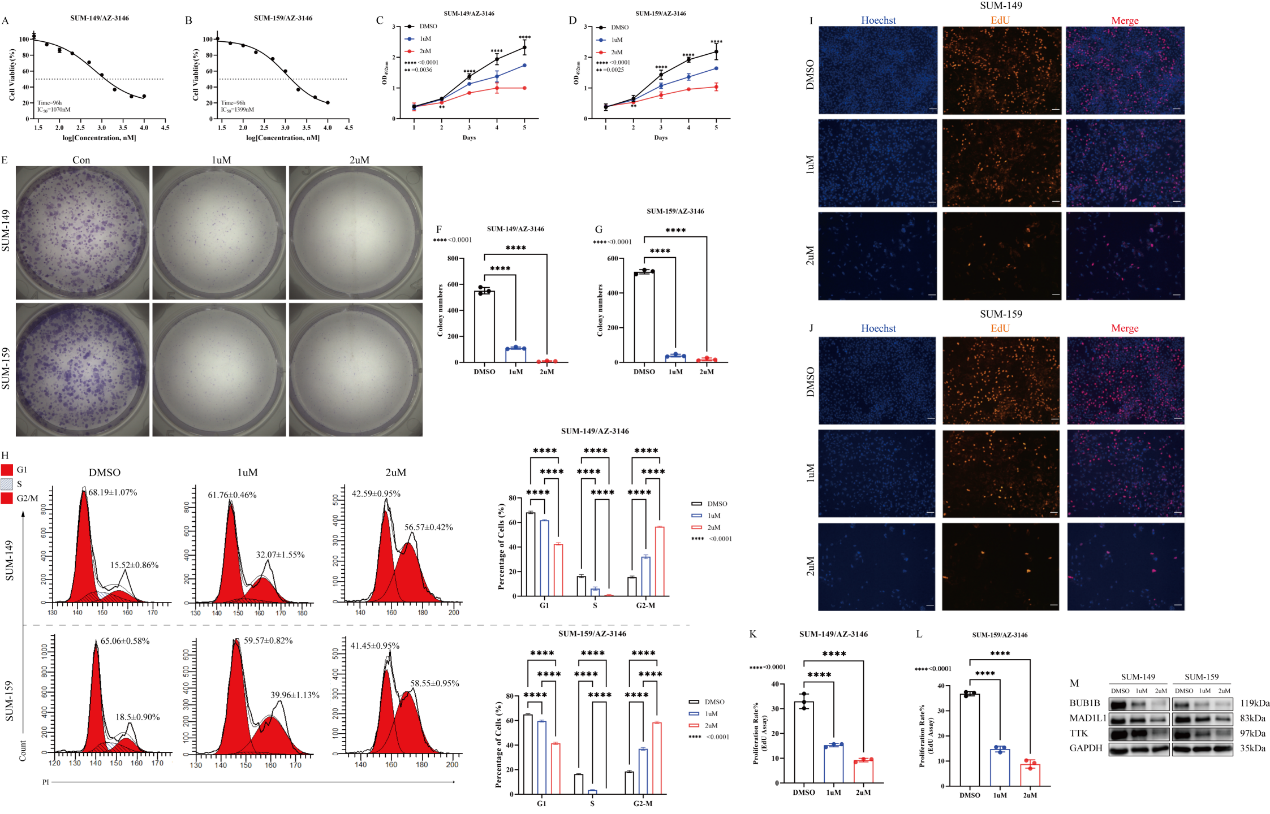


**Supplementary Figure S4.** AZ-3146 inhibits SUM-149 and SUM-159 cell proliferation.

(A, B) IC_50_ values; (C, D) Proliferation curves; (E–G) Changes in colony formation ability; (H) Cell cycle distribution changes; (I–L) Changes in EdU assay; (M) Changes in TTK, MAD1L1, and BUB1B protein expression.


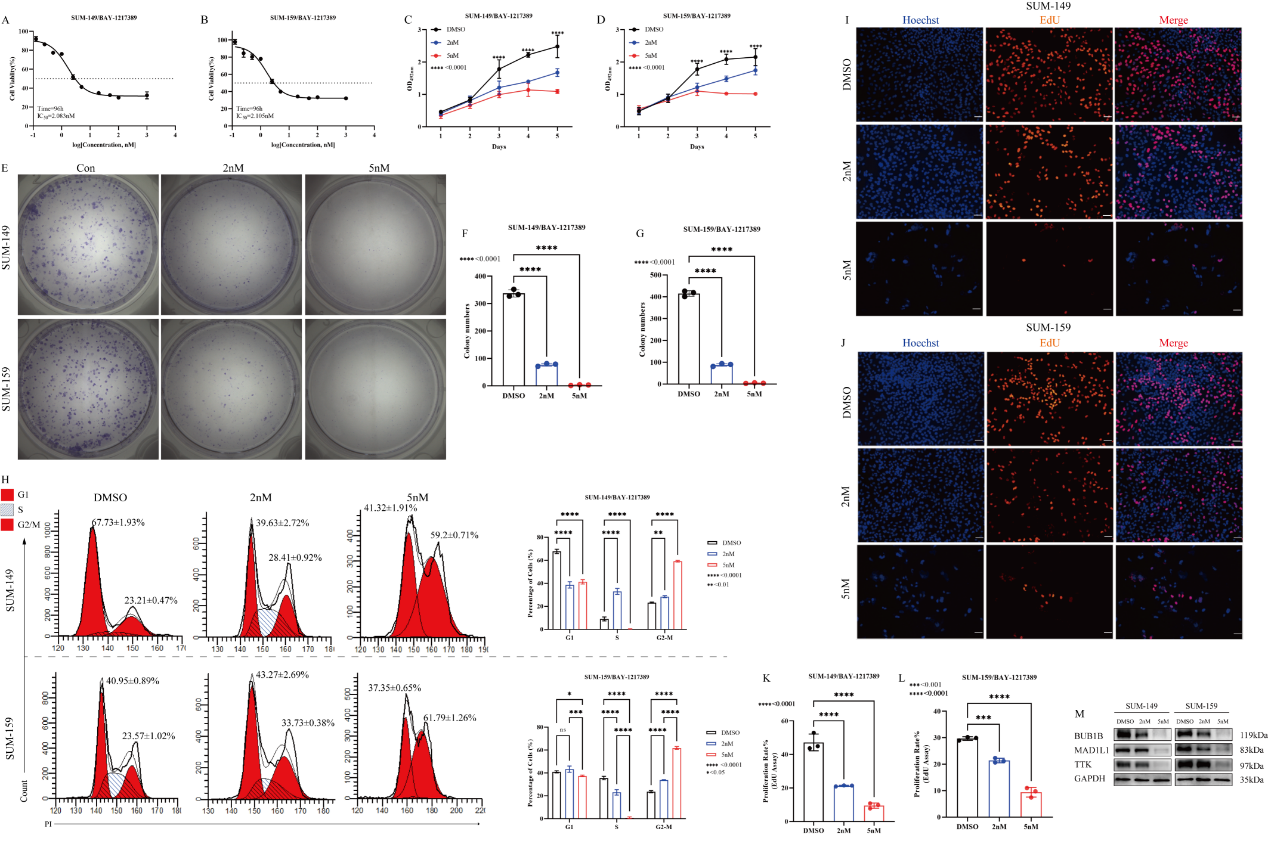


**Supplementary Figure S5.** BAY-1217389 inhibits SUM-149 and SUM-159 cell proliferation.

(A, B) IC_50_ values; (C, D) Proliferation curves; (E–G) Changes in colony formation ability; (H) Cell cycle distribution changes; (I–L) Changes in EdU assay; (M) Changes in TTK, MAD1L1, and BUB1B protein expression.


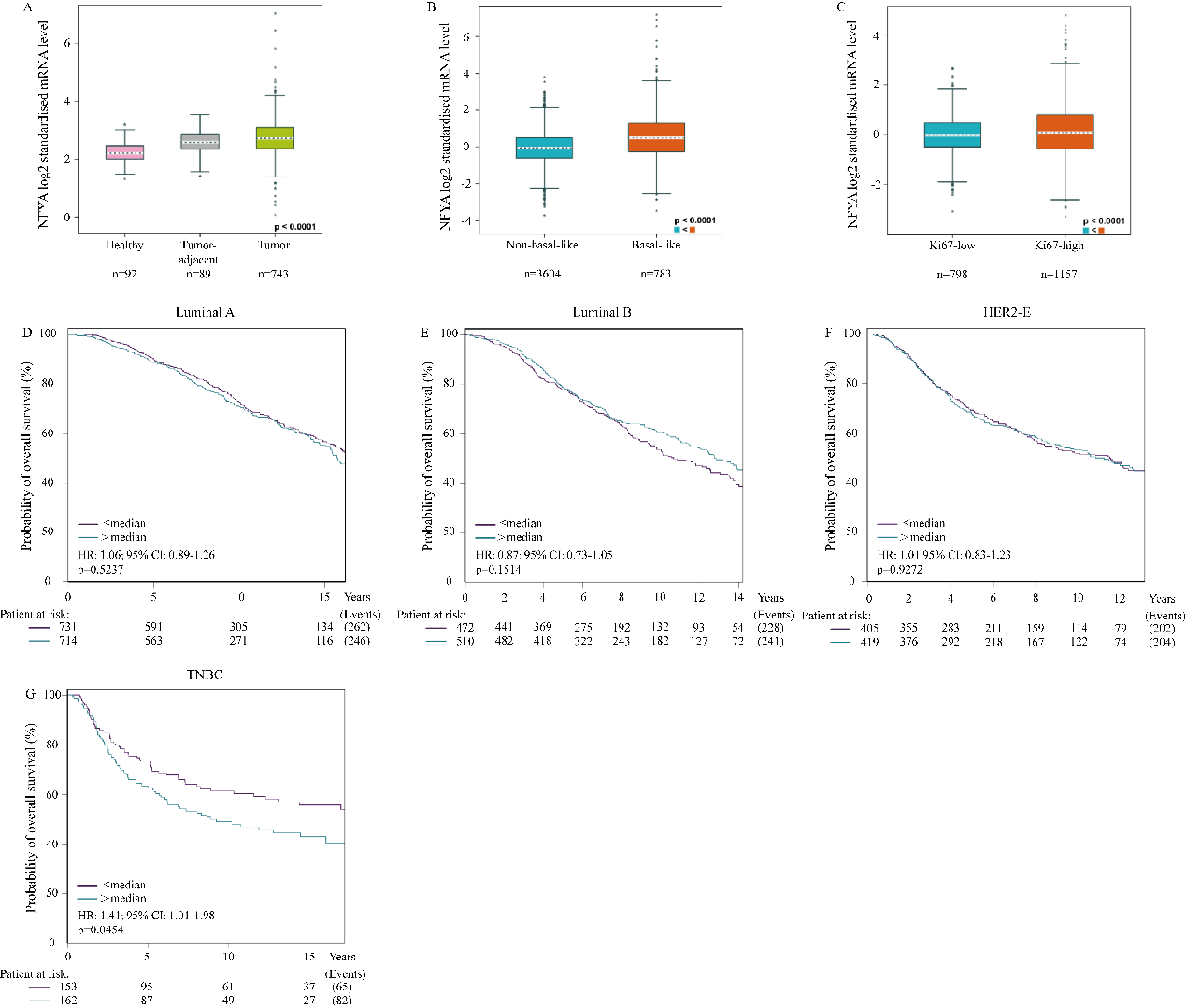


**Supplementary Figure S6.** NFYA is specifically upregulated in TNBC and associated with poor prognosis exclusively in TNBC patients.

(A) NFYA expression in healthy, tumor-adjacent , and tumor tissues; (B) NFYA expression in non-basal-like and basal-like breast cancer; (C) TTK expression across Ki-67 expression subgroups; (D–G) Kaplan-Meier survival curves illustrating overall survival based on TTK expression in various breast cancer subtypes.
