# Supplementary material for: NFYA-Mediated TTK Up-Regulation Drives Fast Cell Cycle Progression and Its Inhibition Leads to Mitotic Catastrophe in Triple Negative Breast Cancer
Source: Cancers (Basel). 2026 Apr 22;18(9):1324. doi: 10.3390/cancers18091324 (PMC13163004; doi:10.3390/cancers18091324)
Supplement: Supplementary file 1 [file cancers-18-01324-s001.zip › The uncropped blots.pdf]

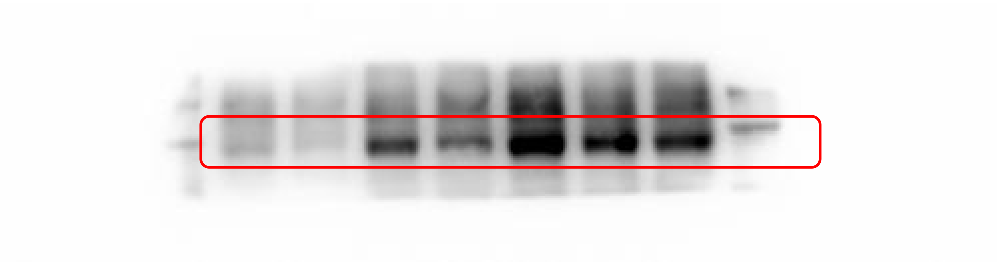

Figure 2__WB-TTK-7-159-L-R

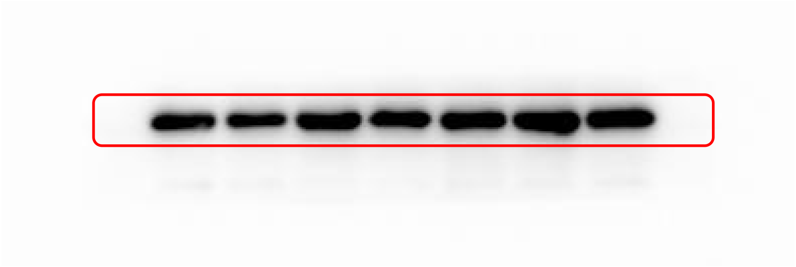

Figure 2_WB-GA-7-159-L-R

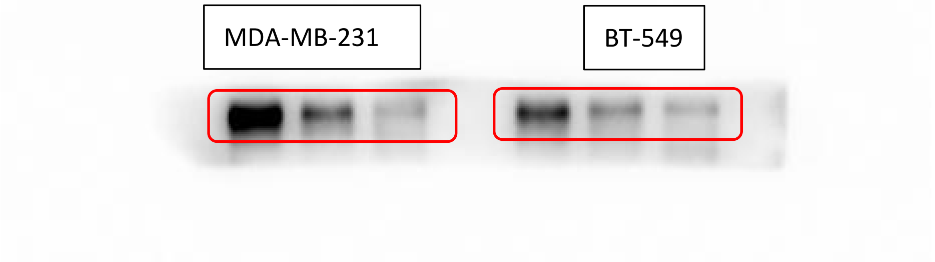

Figure 3-siTTK-BUB1B-231+549

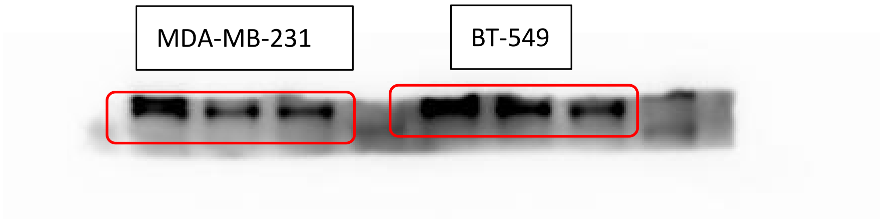

Figure 3-siTTK-MAD1L1-231+549

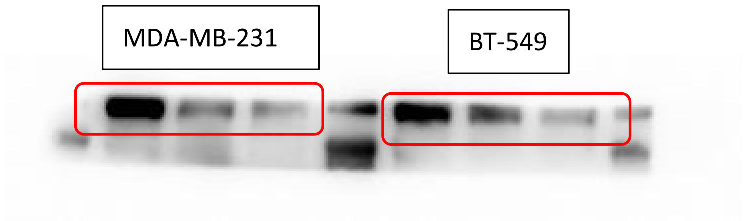

Figure 3-siTTK-TTK-231+549

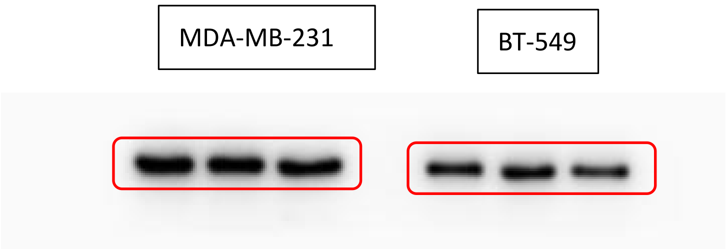

Figure 3-siTTK-GA-231+549

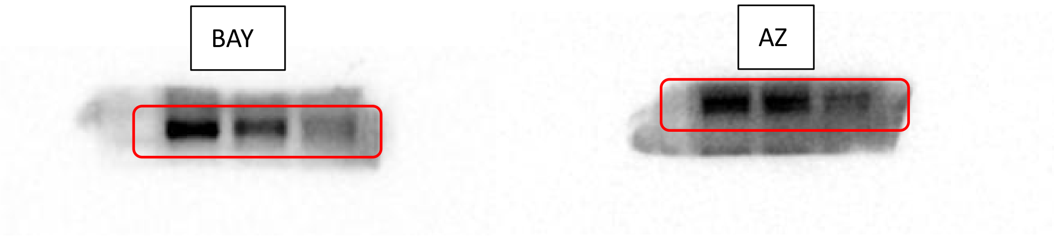

Figure 5-CASP2-231

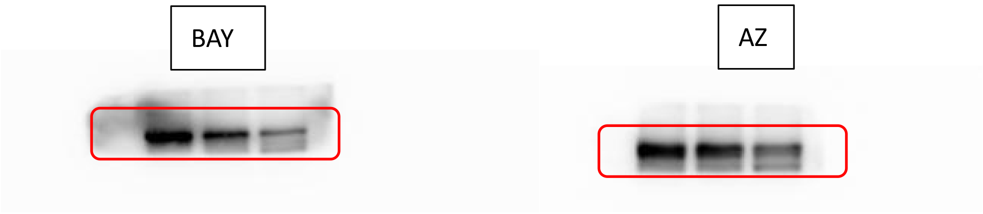

Figure 5-CCNB1-231

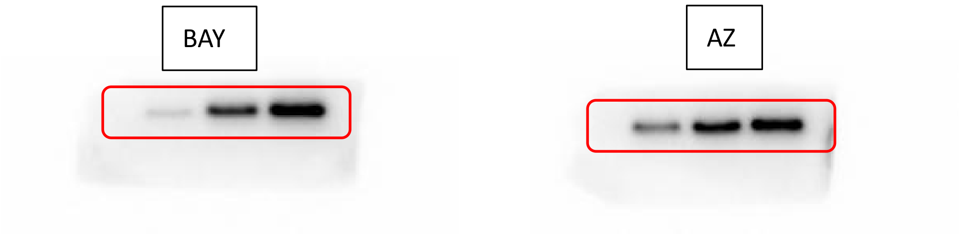

Figure 5-γ-H2AX-231

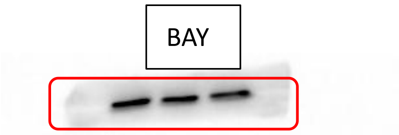

Figure 5-GA-231

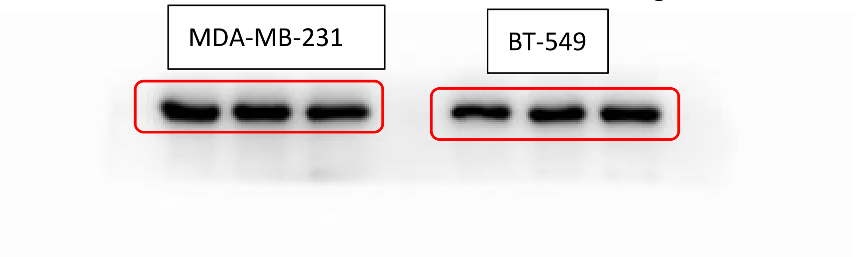

Figure 5-AZ-GA-231+549

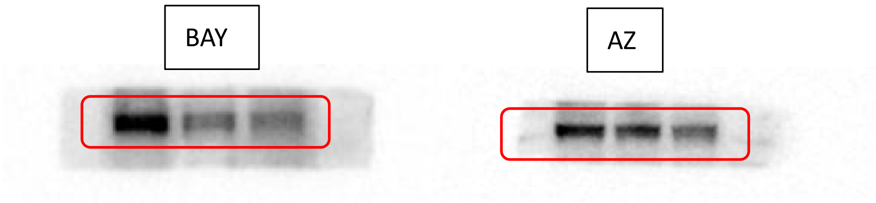

Figure 5-CASP2-549

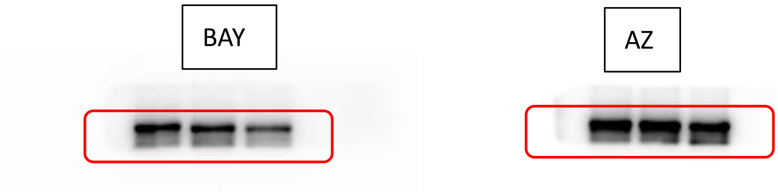

Figure 5-CCNB1-549

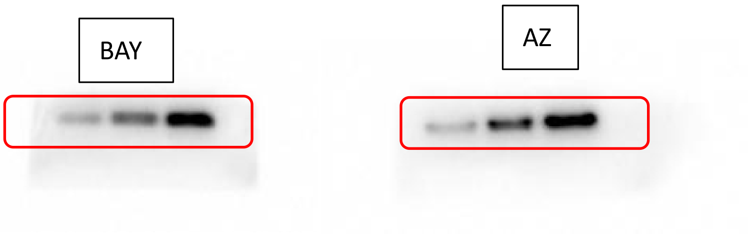

Figure 5-γ-H2AX-549

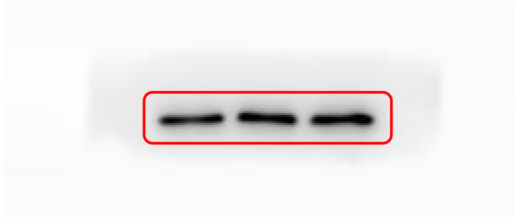

Figure 5-GA-549

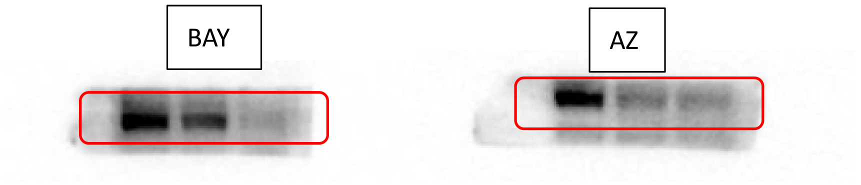

Figure 5-CASP2-149

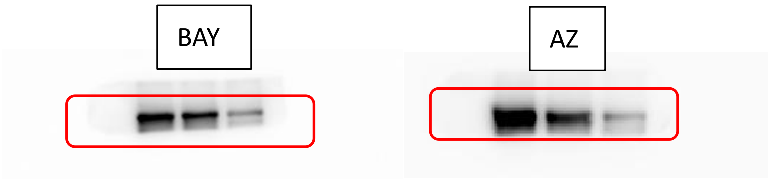

Figure 5-CCNB1-149

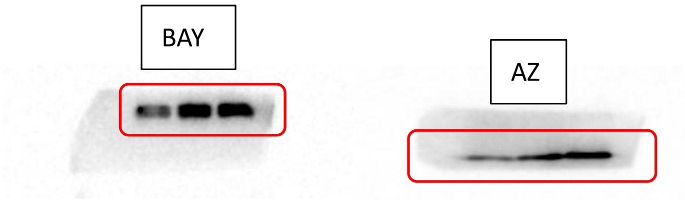

Figure 5-γ-H2AX-149

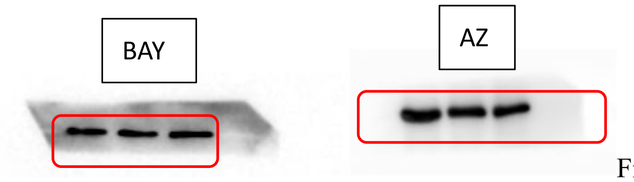

Figure 5-GA-149

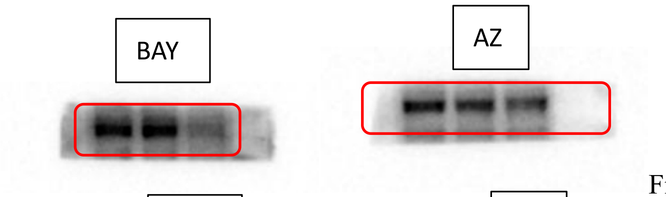

Figure 5-CASP2-159

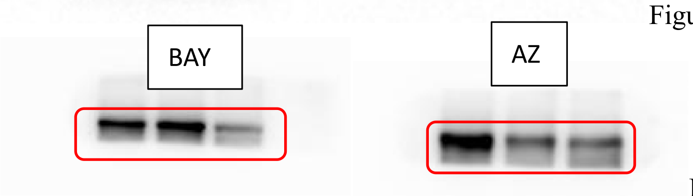

Figure 5-CCNB1-159

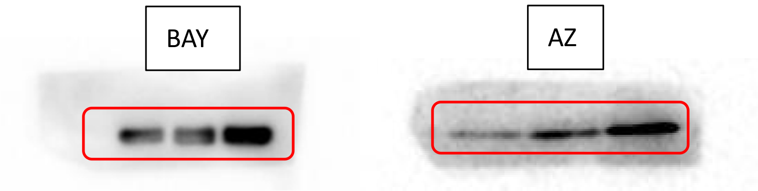

Figure 5-γ-H2AX-159

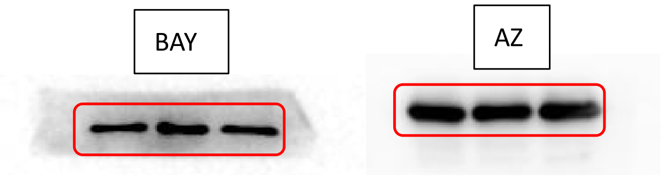

Figure 5-GA-159

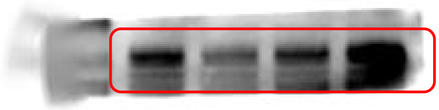

Figure 6-TTK-siNFYA+OENFYA

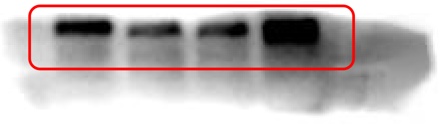

Figure 6-NFYA-siNFYA+OENFYA

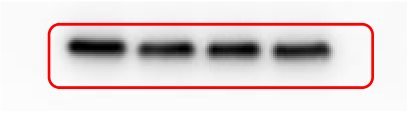

Figure 6-J-GA

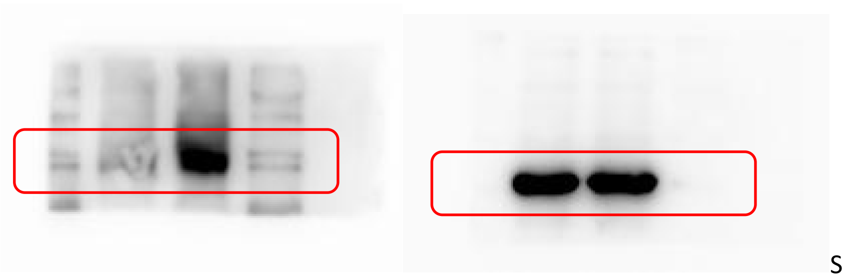

SF. 1-1-TTK+GA

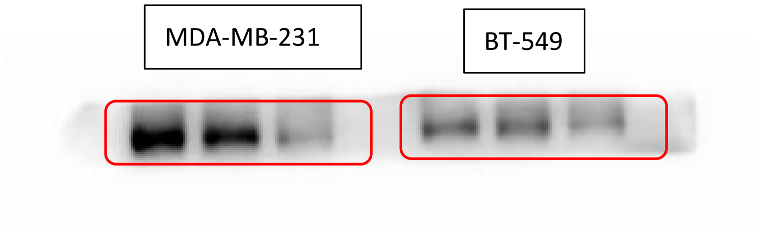

SF. 2--BUB1B-AZ-231+549

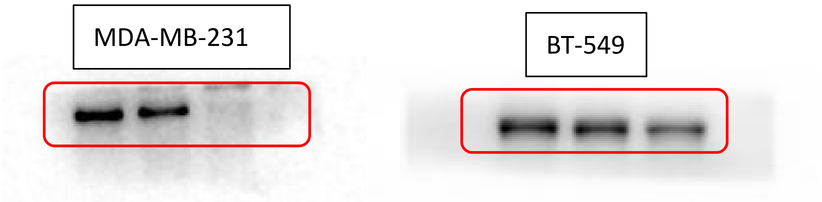

SF. 2--MAD1L1-AZ-231+549

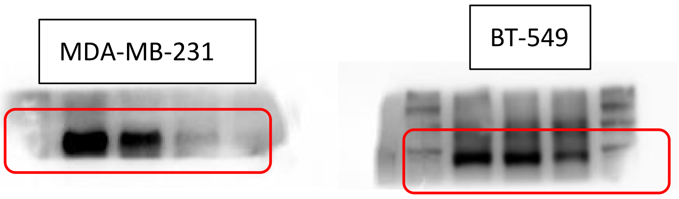

SF. 2--TTK-AZ-231+549

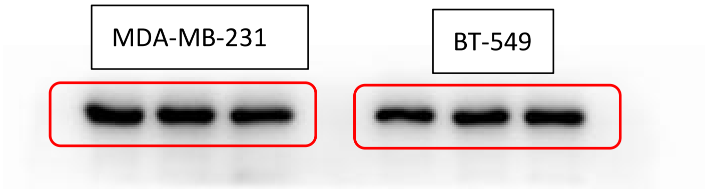

SF. 2-GA-AZ-231+549

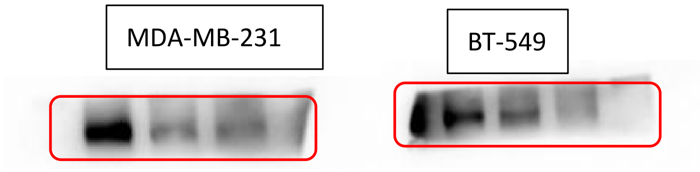

SF. 3-BUB1B-BAY

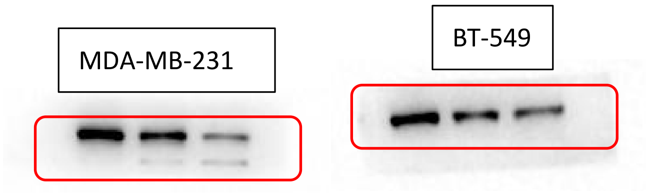

SF. 3-MAD1L1-BAY

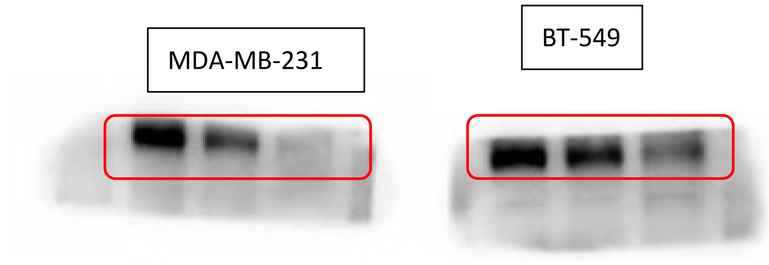

SF. 3-TTK-BAY

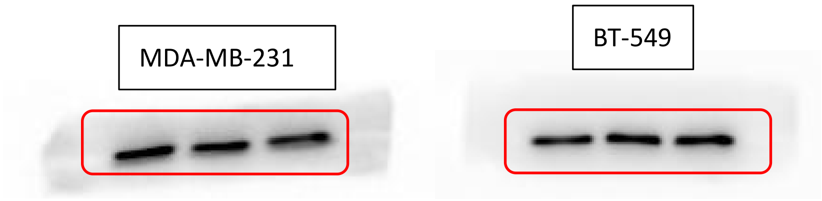

SF. 3-GA-BAY

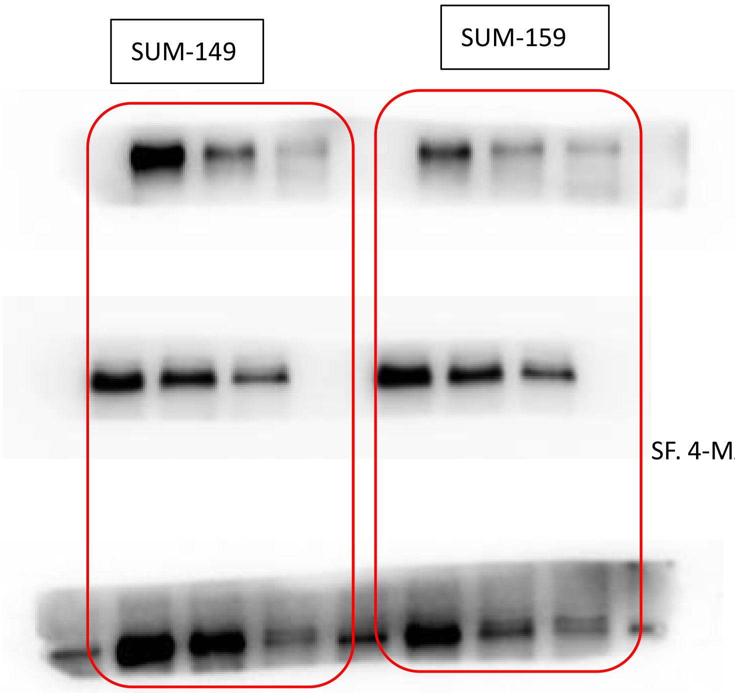

SF. 4-BUB1B-AZ

SF. 4-MAD1L1-AZ

SF. 4-TTK-AZ

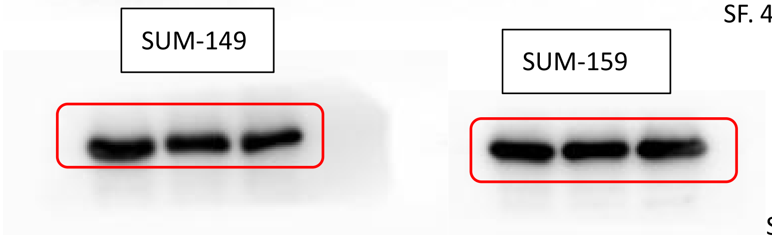

SF. 4-GA-AZ

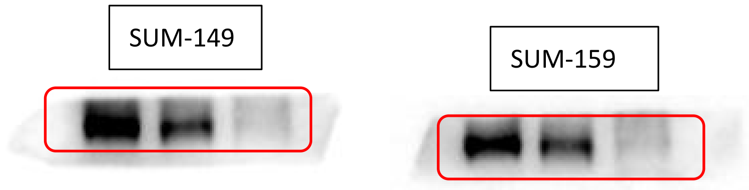

SF. 5-BUB1B-BAY

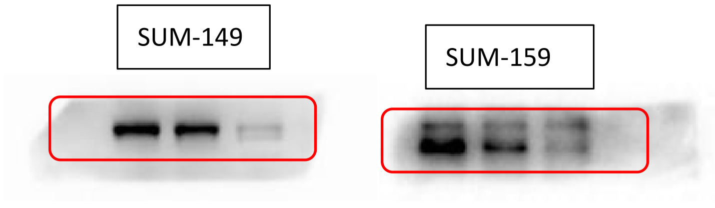

SF. 5-MAD1L1-BAY

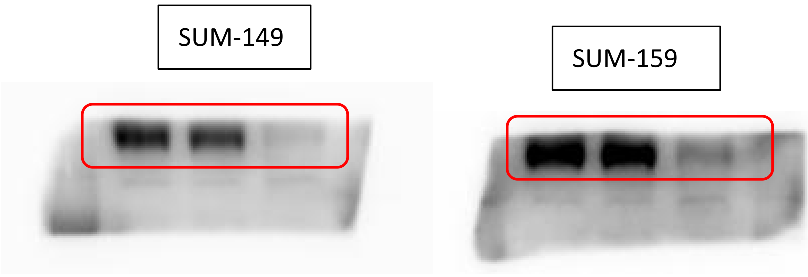

SF. 5-TTK-BAY

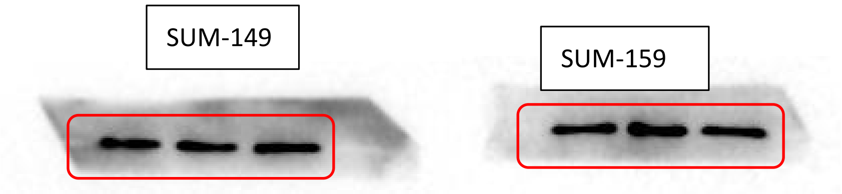

SF. 5-GA-BAY
